# The American Academy of Medicine

**Published:** 1894-09

**Authors:** 


### THE AMERICAN ACADEMY OF MEDICINE.

An interesting and profitable meeting of this body was held on the 29th and 30th of August, at Jefferson, N. H., under the presidency of Dr. George M. Gould, of Philadelphia. It is generally known that this organization consists of graduates of literary institutes, who have the degree of A.B. or A.M., and who have also received the degree of M.D. But it is not perhaps so well understood that the scope of investigation undertaken by this Society does not include the practical details which occupy other medical associations, and that pathology and therapeutics are outside of its sphere of operations. Originally its work was confined chiefly to a consideration of the means of improving the requisites for entering upon the study of medicine and enlarging the domain of medical education in our schools. In a quiet way its impress has gone forth to the profession from year to year during nearly a quarter of a century, and doubtless some of the advances which are embodied in the action of the Association of American Medical Colleges held at San Francisco, June 7, 1894, are due in part to the steady and persistent claim of the American Academy of Medicine for higher medical education. The same will hold good in regard to the onward and upward movement of the Southern Medical College Association in regard to preliminary requirements and a longer period of study in the curriculum of the different medical schools.

There is one line upon which I trust that the Academy may bring its influence to bear in the future, and that is in favor of requiring students of medicine to conform to the requirements of former days by systematic attention to the elementary principles under a preceptor, or under a special instructor, before attending lectures in a medical college. It matters not what proficiency the student may have in mere literary training, if he has not obtained such knowledge as is only to be found in the elementary text-books, he will be incapable of appreciating the courses of lectures in our medical colleges.

The extension of the scope for investigation by the Academy to questions of medical sociology, and to matters which bring the medical profession into relation with the organization of institutes for bettering the condition of certain classes, was indicated at the recent meeting in a definite manner. The discussion of charities as related to medicine by the President, and the subject of blindness in connection with its influence upon society generally, along with other important topics of interest presented in the several papers, manifested a fixed purpose on the part of the Academy to cut loose from the beaten path of educational matters. At the next meeting it is purposed to open up wider fields of inquiry, and to investigate lunacy in all its phases, as well as epilepsy, and other kindred subjects bearing upon the mental relations of the profession and the people in solving these problems.

The elevated plane occupied by the Academy appeals to all who are eligible for membership to cast their lot with it. There were thirty-one new fellows added at the recent meeting, and it is hoped that a large accession from the South may swell the list at the forthcoming meeting in Baltimore. J. M'F. G.

